# A multi-antigenic MVA vaccine increases efficacy of combination chemotherapy against *Mycobacterium tuberculosis*

**DOI:** 10.1371/journal.pone.0196815

**Published:** 2018-05-02

**Authors:** Stéphane Leung-Theung-Long, Charles-Antoine Coupet, Marie Gouanvic, Doris Schmitt, Aurélie Ray, Chantal Hoffmann, Huguette Schultz, Sandeep Tyagi, Heena Soni, Paul J. Converse, Lilibeth Arias, Patricia Kleinpeter, Benoît Sansas, Khisimuzi Mdluli, Cristina Vilaplana, Pere-Joan Cardona, Eric Nuermberger, Jean-Baptiste Marchand, Nathalie Silvestre, Geneviève Inchauspé

**Affiliations:** 1 Transgene SA, Infectious Diseases department, Centre d’Infectiologie, Bât Domilyon, Lyon, France; 2 Transgene SA, Boulevard Gonthier d’Andernach, Parc d’innovation, Illkirch-Graffenstaden, France; 3 Center for Tuberculosis Research, Department of Medicine, Johns Hopkins University, Baltimore, Maryland, United States of America; 4 Unitat de Tuberculosi Experimental, Fundació Institut Germans Trias i Pujol, Universitat Autònoma de Barcelona, CIBERES, Crta Badalona, Catalonia, Spain; 5 Global Alliance for Tuberculosis Drug Development, New York, New York, United States of America; Colorado State University, UNITED STATES

## Abstract

Despite the existence of the prophylactic Bacille Calmette-Guérin (BCG) vaccine, infection by *Mycobacterium tuberculosis* (Mtb) remains a major public health issue causing up to 1.8 million annual deaths worldwide. Increasing prevalence of Mtb strains resistant to antibiotics represents an urgent threat for global health that has prompted a search for alternative treatment regimens not subject to development of resistance. Immunotherapy constitutes a promising approach to improving current antibiotic treatments through engagement of the host’s immune system. We designed a multi-antigenic and multiphasic vaccine, based on the Modified Vaccinia Ankara (MVA) virus, denoted MVATG18598, which expresses ten antigens classically described as representative of each of different phases of Mtb infection. *In vitro* analysis coupled with multiple-passage evaluation demonstrated that this vaccine is genetically stable, *i*.*e*. fit for manufacturing. Using different mouse strains, we show that MVATG18598 vaccination results in both Th1-associated T-cell responses and cytolytic activity, targeting all 10 vaccine-expressed Mtb antigens. In chronic post-exposure mouse models, MVATG18598 vaccination in combination with an antibiotic regimen decreases the bacterial burden in the lungs of infected mice, compared with chemotherapy alone, and is associated with long-lasting antigen-specific Th1-type T cell and antibody responses. In one model, co-treatment with MVATG18598 prevented relapse of the disease after treatment completion, an important clinical goal. Overall, results demonstrate the capacity of the therapeutic MVATG18598 vaccine to improve efficacy of chemotherapy against TB. These data support further development of this novel immunotherapeutic in the treatment of Mtb infections.

## Introduction

Tuberculosis (TB) continues to be a major global health issue. In 2015, 1.8 million infected persons died from the disease and 10.4 million new cases of TB were reported worldwide [1]. TB is primarily a pulmonary disease caused by aerosol infection with *Mycobacterium tuberculosis* (Mtb). Only 5% of infected individuals develop an active form of the disease whereas for the remaining 95%, adaptive cellular immunity can contain the infection, resulting in so-called latent tuberculosis infection (LTBI), during which bacteria are believed to survive in a non-replicating stage inside granulomas [2]. Long-term latent TB infection can give rise to reactivation in an estimated 10% of cases. In low-endemic regions, reactivation of LTBI seems to be the main source of TB disease in the adult population whereas reinfection is more likely to happen in high burden areas [3, 4].

The only approved prophylactic vaccine is the attenuated *Mycobacterium bovis*-based Bacille Calmette-Guérin (BCG) vaccine. It is efficient against severe forms of TB in children, but provides variable efficacy against adult pulmonary TB [5]. Standard of care treatment of active TB patients is currently based on combination chemotherapy that displays various degrees of efficacy depending on the infecting strain. Although patients infected with a drug-sensitive (DS) Mtb strain are efficiently cured by a 6-month first-line antibiotic regimen, low compliance to the long treatment duration remains a major cause of drug-resistance acquisition by mycobacteria. A second line antibiotic regimen is given to treat multi-drug resistant (MDR) TB patients during a period that can last up to 24 months and which displays variable efficacy (often less than 60%) and is associated with severe adverse effects and poor outcomes. Finally, patients infected with extensively drug-resistant (XDR) Mtb strains which are not sensitive to the two first classes of antibiotics are treated with third-line drug regimens leading to very poor efficacy (<10%) during a period exceeding 2 years. Emergence of drug resistance constitutes a global health issue. Although the worldwide prevalence of MDR-TB is assessed at 5%, this varies from approximately 1% to more than 20% according to regions of the Globe [1, 2]. Furthermore, XDR- and TDR (totally drug-resistant)-TB have emerged worldwide as a threat to public health and TB control, raising concerns about a possible future epidemic of virtually untreatable TB. The frequency and distribution of MDR-TB cases is described by the American Centers for Disease Control (CDC) as ranging from 3–5% of the confirmed TB cases in the US, XDR-TB representing up to 4% of these cases.

Efforts are ongoing to develop new antibiotics and novel drug regimens appear to shorten under some conditions MDR treatment to 9 months [6]. Yet, this strategy will inevitably be associated with the development of antibacterial resistance. Resistance to the most recent new TB drugs entering the market, namely bedaquiline and delamanid, has already been reported [7]. In contrast, strategies aiming at harnessing patient’s immune response against Mtb may protect actively infected individuals against disease progression and might accelerate cure and/or increase cure rate obtained by antibiotic therapy. An antigen-specific vaccine-based immunotherapy should be capable to optimize the infected host-defective Th1-directed response resulting in regain of immune control and definite elimination of the bacilli, and in particular of non-replicating bacilli [8–10]. This concept is gaining increasing interest and a few candidate therapeutic vaccines aiming at improving treatment of active tuberculosis have shown safety and early promising preclinical results. These vaccines have relied on recombinant adjuvanted proteins or inactivated Mtb strains [11–14]. Most of them have been evaluated in chronic post-exposure mouse models *i*.*e*. they have typically been applied in combination with suboptimal antibiotic regimens in mice [15] and have shown capacity to limit the regrowth of persisting Mtb bacilli load after the discontinuation of treatment. Clearly caution will be required to avoid detrimental immune-linked effects such as exacerbation of inflammation and immune therapeutics are likely to be best placed in contexts lowering first bacterial loads *i*.*e*. post-initiation of antibiotic treatment.

While no correlates of protection and/or infection control have yet been fully identified after exposure to Mtb, it is generally agreed that T-cell immunity plays a role [16]. The first generation of TB therapeutic vaccines have mainly relied on platforms that are mostly optimal for induction of B-cell-based immunity (*e*.*g*. adjuvanted recombinant proteins). Hence, it appears very attractive to direct efforts to platforms favoring cellular-based immunity such as “vectored-vaccines”, in particular based on safe, non-propagative viruses. The poxvirus Modified Vaccinia Ankara (MVA) is a well-established vaccine platform that is particularly efficient at induction of immunogen-specific T cells (CD4 and/or CD8 responses) [17]. It harbors a remarkable plasticity allowing to encode large immunogenic sequences (up to 10 kb or more). In addition, its well-established safety profile, combined with the fact that it has already been tested in therapeutic clinical trial settings up to phase 2 [18], make it a vector of choice for immunotherapeutic evaluation against tuberculosis. We have earlier described the value of MVA in the development of a highly immunogenic multiphasic tuberculosis vaccine candidate [19]. We report here the production of a genetically stable MVA therapeutic vaccine expressing 10 Mtb antigens representative of different phases of infection, documented in *in vivo* and/or *in vitro* replication models representative of commonly described active, latent and resuscitation phases. We show that this vaccine promotes Mtb antigen-specific Th1 response through production of IFNγ and does trigger specific cell cytolysis upon vaccination of mice. Using two mouse post-exposure models, we demonstrate that this novel MVA vaccine is an effective adjunct to antibiotic therapy to treat TB.

## Material and methods

### Mice

For immunogenicity experiments, six to eight weeks old female BALB/c, C57BL/6 and CB6F1 hybrid mice were purchased from Charles River Laboratories (L’Arbresle, France) and housed at Plateau de Biologie Expérimentale de la Souris (PBES, Lyon, France) in ventilated cages. The mouse experiments were carried out in accordance with the animal care guidelines of the European Union and French laws and were approved by the local Animal Ethic Evaluation Committee CECCAPP (Comité d’Evaluation Commun au Centre Léon Bérard, à l’Animalerie de transit de l’ENS, au PBES et au laboratoire P4) under the following protocol numbers: ENS-2012-028 and ENS-2014-039.

For efficacy experiments, female BALB/c and C57BL/6 mice 5–6 weeks of age were obtained from Charles River Laboratories (Wilmington, MA, USA; n = 12–15 per experimental group) and Envigo (Sant Feliu de Codines, Catalonia, Spain; n = 12 per experimental group), respectively. All animal procedures were approved by the Animal Care and Use Committee of Johns Hopkins University and Animal Experimentation Ethics Committee of the Hospital Universitari Germans Trias i Pujol under the protocol numbers MO13M33 and MO15M325, and DAAM7896, respectively.

At the end of experiments, mice were sacrificed either by cervical dislocation or by cardiac puncture on mice anesthetized with ketamine (100 mg/kg) and xylazine (10 mg/kg).

### Construction of MVATG18598

The MVA-based MVATG18598 virus encodes 10 Mtb antigens expressed as 5 protein fusions: Rv2626/T2A/Ag85B, CFP10/ESAT6, TB10.4/Rv0287, RpfB-RpfD and Rv3407/E2A/Rv1813. Some of these Mtb antigens are prone to associating and forming a 1:1 complex (heterodimer) during natural infection. The formation of such heterodimers was demonstrated for ESAT6 and CFP10 as well as TB10.4 and Rv0287 [20]. Therefore, both partners were fused together to improve the expression and/or the stability of the corresponding proteins. Moreover, fusions of 2 unrelated mycobacterial antigens were also performed in order to express 10 different antigens in the same MVA (under the control of 5 promoters). In some fusions, sequences coding for self-cleaving 2A peptides were added between the two elements of the fusion to allow the synthesis of both components separately. 2A peptide mediates a co-translational cleavage at its own C-terminus and is proposed to cause the ribosome to skip the synthesis of a specific peptide bond, producing a discontinuity in the peptide backbone [21]. For some proteins, wild-type sequence (TB Database, www.tbdb.org) has been conserved, deleted or mutated. The first fusion Rv2626/T2A/Ag85B was constituted by the full-length Rv2626 (full length wild type sequence) protein followed by a GSG linker and fused to the T2A peptide from *Thosea asigna* virus and the residues 33 to 325 of Ag85B polypeptide (wild type sequence without a part of signal peptide). The second fusion, CFP10/ESAT6, was constituted by the full-length CFP10 protein fused to the full-length ESAT6 protein separated by a linker composed of residues 149 to 162 of the Mtb protein Rv1827. The third fusion, TB10.4/Rv0287, was constituted by the full-length TB10.4 protein fused to the full-length Rv0287 protein separated by a linker composed of residues 149 to 161 of the Mtb protein Rv1827. The fourth fusion, RpfB-RpfD was constituted by the residues 30 to 284 of RpfB (*i*.*e*. N-terminal part of the protein without its predicted signal peptide) fused to residues 54 to 154 of mutated RpfD (E61K, T84A and Q113A). The fifth fusion, Rv3407/E2A/Rv1813 was constituted by the full-length Rv3407 protein followed by a GSG linker and fused to the E2A peptide from *Equine rhinitis* A virus and the residues 31 to 143 of Rv1813 polypeptide (*i*.*e*. wild type protein without the predicted signal peptide).

Synthetic genes coding for the different Mtb fusions were generated by Geneart (Regensburg, Germany). The sequences were optimized for human codon usage, a Kozak sequence (ACC) was added before the ATG starting codon and a 3’TTTTTNT transcriptional terminator sequence was added immediately after the stop codon.

The fusion Rv2626/T2A/Ag85B was placed under the control of the pB2R promoter while the fusion CFP10/ESAT6 was inserted downstream the pH5R promoter [22]. The fusion TB10.4/Rv0287 was placed under the control of pSE/L promoter [23] while the fusion RpfB-RpfD was inserted downstream the p7.5K promoter [24] and the fusion Rv3407/E2A/Rv1813 was placed under the control of pA35R promoter.

To create recombinant vaccinia virus, pTG17960 transfer vector allowing homologous recombination in the so-called deletion III corresponding site of the MVA genome was used [25]. The five expression fusions were cloned as a head-to-tail concatemer into the pTG17960 transfer vector by standard cloning techniques.

Recombinant MVA was then generated by standard procedures as described previously [19] by transfecting the transfer plasmid into MVA infected primary chicken embryo fibroblasts (CEF). After construction of the virus, gene expression, sequence of inserted DNA, and viral purity were verified. The absence of parental virus was checked by PCR analysis.

After 10 serial passages in CEF in F175 tissue culture flask, genetic stability was assessed on 100 individual clones by PCR and Western-blot analysis.

### *In vitro* expression

For analysis of expression, CEF cells were mock-infected with MVATGN33.1 (control empty MVA) or infected with MVATG18598 at a multiplicity of infection of 0.2. For detection of Rv3407/E2A/Rv1813 fusion, MG132 (proteasome inhibitor, 10 μM) was added 30 min post-infection. Cells were lysed 24 h post-infection. Infected cell lysates were subjected to SDS-PAGE (4–15% Criterion^TM^ TGX^TM^ gels, Biorad) and the separated proteins were transferred to PVDF membranes for immunoblotting. The following primary antibodies were used for the detection of mycobacterial fusions: monoclonal antibody for ESAT6 (clone HYB076-08, Santa-Cruz); monoclonal antibody for Rv2626 (clone 26A11, Lifespan-Biosciences), rabbit polyclonal anti-serum for Ag85B (NR-13800, BEI), rabbit polyclonal antibody for TB10.4 (ABIN361292, Antibodies-online) and polyclonal rabbit antibodies for Rv3407, RpfB and Rv1813 (generated by Eurogentec, Seraing, Belgium). Goat anti-mouse or anti-rabbit HRP-conjugated antibodies (DakoCytomation) were used as secondary antibody and immune-complexes were detected using the Amersham ECL prime Western blotting (GE Healthcare).

### Immunization

For immunogenicity studies in naïve mice, each mouse was injected subcutaneously (sc) with 1x10^7^ plaque-forming units (pfu) in 100 μL of Tris-HCl saccharose buffer. MVA was injected once or twice 2 weeks apart depending on the immune analysis performed afterwards and the assay performed 7 days after last injection.

### Peptides

*In vitro* stimulations of splenocytes were performed using a synthetic peptide library (ProImmune) comprised of 15-mer peptides overlapping by 11 residues as described previously [19]. For each antigen, peptide pools contained up to 25 peptides. Therefore, depending on the length of a given antigen, 1 to 4 pools were required to cover its full sequence (S1 Protocol). An irrelevant β-Gal peptide (10 μM) or pool of peptides covering β-Gal sequence was used as negative control in ELISpot assays.

### IFNγ ELISpot assay

IFNγ response was monitored one week after one sc injection of MVA (10^7^ pfu/mouse in 100 μL of Tris-HCl saccharose buffer). IFNγ ELISpot assays were performed as previously described [26]. Briefly, splenocytes were stimulated *in vitro* with pools of peptides at 1 μM for 40 h in presence of IL2. Spots were quantified using an ELISpot microplate reader (CTL-Europe GmbH, Germany). Results are represented as the mean value obtained for triplicate wells reported to 10^6^ cells. Threshold of positivity was defined as the mean value obtained in unstimulated condition plus 2 standard deviations (SD). Statistical analyses (R (3.0.2) software) were performed using a Kruskal-Wallis test followed if significant (*p*<0.05) by pairwise Mann-Whitney tests to compare group by group. The difference observed between two groups was considered as significant if the *p*-value obtained with the Mann-Whitney test is lower than 0.05.

### *In vivo* cytotoxic T lymphocyte (CTL) assay

*In vivo* CTL assays were performed as previously described [19]. We selected CB6F1 mice derived from crossing of BALB/c and C57BL/6 mice. Mice (at least 5 mice per group) were vaccinated twice at 2-week interval with either MVATGN33.1 or MVATG18598 candidate and the CTL assay was performed one week after last injection. Briefly, peptide-pulsed splenocytes from naive mice were labelled with different concentrations of carboxyfluorescein succinimidyl ester (CFSE, Molecular Probes) and/or CellTrace^TM^ Violet (CTV, Molecular Probes), and injected intravenously in vaccinated mice or unvaccinated naïve mice. After 20 h, splenocytes from recipient mice were analyzed by flow cytometry. Percentage of specific killing was determined by the following formula: % of specific lysis = [100 − (R_MVATG18598 or MVATGN33.1_ / R_Naïve_)] × 100 where R = (number of peptide-pulsed target cells / number of unpulsed target cells). Statistical comparisons between groups immunized with MVATG18598 and MVATGN33.1 were performed using permutation resampling test applied on ratios (R_MVATG18598_ and R_MVATGN33.1_). *p* value less than or equal to 0.05 were considered as significant. Analyses were performed using R version 3.0.2. For each peptide pool, results are represented as the percentage of lysis obtained with the MVATG18598 minus the percentage of lysis obtained with the MVATGN33.1.

### Cytokine assay

Spleens of Mtb-infected BALB/c mice were harvested and splenocytes prepared as previously described [26]. Briefly, spleen cells (5.10^5^/well) of individual mice were stimulated with 1 μM of complete peptide pools covering TB10.4, RpfB-RpfD and Rv2626 protein sequences for 48 h at 37°C. To monitor T cell responses specific to the MVA vector, a 9-mer peptide belonging to MVA vector was also used. Supernatants were then harvested, 0.22 μm-filtered and stored at -20°C until use. The day of the cytokine assay, supernatants were allowed to thaw at RT, diluted at a 1:2 ratio with complete medium, and 25 μL of each diluted sample were assayed using the Millipex MAP Mouse Th17 magnetic bead panel kit (Millipore) following the manufacturer’s instructions. Results were then analyzed using Bio-Plex MAGPIX reader (Biorad). Controls with cells cultured in medium alone were included, and the background cytokine production from these cultures subtracted. A first statistical analysis was done using a Kruskal-Wallis test. If a significant difference was found, pairwise Mann-Whitney tests were performed.

### Enzyme-linked immunosorbent assay (ELISA)

Blood samples were collected by cardiac puncture on anesthetized mice (ketamine, 100 mg/kg and xylazine, 10 mg/kg) as a terminal procedure at the end of post-exposure experiments. Blood samples were centrifuged twice at 4000 rpm for 5 min at 4°C, and serum-containing supernatants were stored at -20°C until use.

Briefly, all coated antigen proteins were prepared as follows. A six-histidine (His) tag was fused to either N- or C-terminus of each antigen which was produced as recombinant protein in *E*. *coli* and purified by affinity chromatography on immobilized nickel resin according to provider instructions (Qiagen). For preparation of heterodimers ESAT6/CFP10 and TB10.4/Rv0287, each protein was produced and purified independently as described above. The His tag of CFP10 and Rv0287 was removed by thrombin cleavage and 1.5 to 2 molar excess of these proteins was added to their respective His-tagged partner (*i*.*e*. ESAT6 and TB10.4, respectively) and incubated at room temperature for 2 hours. The excess of untagged proteins was removed by loading the mix on nickel resin and the dimers were eluted by imidazole. Dimers were further purified by preparative size exclusion chromatography equilibrated in PBS.

In a 96-well plate (Maxisorp Nunc), 100 μL/well of immobilization buffer (0.05 M Carbonate, pH 9.6) containing different concentrations of each antigen were incubated overnight at 4°C. Plates were then washed 3 times with 300 μL of wash buffer (20 mM Tris pH 7.5, 150 mM NaCl, 0.05% Tween20) and blocking buffer (200 μL/well, wash buffer + 5% non-fat dry milk (Biorad)) was added. Plates were incubated 1 h at room temperature and washed 3 times with 300 μL/well of wash buffer. Two-fold serial dilutions of samples starting at 1/50 in blocking buffer were applied (100 μL final volume). Plates were then incubated 1 h at 37°C and washed. In each well, 100 μL of a 1/5000 dilution of polyclonal anti-mouse IgG+IgM+IgA-heavy and light chains HRP goat antibody (Bethyl, 100 ng/mL final) in blocking buffer were added and plates were incubated 1 h at 37°C and then washed. 3,3’,5,5’-Tetramethylbenzidine (TMB) solution (100μL/well, Sigma) was added and plates were incubated 30 min at 37°C. Reaction was stopped by addition of 100 μL/well of 2 M sulfuric acid and absorbance was measured at 450 nm using a plate reader (Tecan IF200). Absorbance was plotted versus the log_10_ of the dilution factor. Endpoint titer was determined to be the reciprocal of the highest serum dilution at which the OD value was equal to or greater than ten times the mean value of the absorbance obtained without serum. As serum sample dilution started at 1/50, all titer values below 1.69 were considered as null. Statistical analyses (R (3.0.2) software) were performed using a Kruskal-Wallis test followed if significant (*p*<0.05) by pairwise Mann-Whitney tests to compare group by group. The difference observed between two groups was considered as significant if the *p*-value obtained with the Mann-Whitney test is lower than 0.05.

### Post-exposure models

BALB/c mice were infected with *M*. *tuberculosis* H37Rv (ATCC 27294), using the Inhalation Exposure System (Glas-Col, Terre Haute, IN, USA) and a fresh log-phase broth culture (optical density at 600 nm, 0.8 to 1.0), diluted to implant approximately 2.0 log_10_ CFU in the lungs of each mouse, as previously described [27]. After the peak of infection (4 weeks post inoculation), mice (12–15 animals/group) started treatment with an antibiotic regimen (RHZ), composed of rifampin (R, 10 mg/kg), isoniazid (H, 10 mg/kg) and pyrazinamide (Z, 150 mg/kg), daily five days per week. After 8 weeks of treatment, Z was discontinued, and treatment with RH continued for 3 additional weeks, until CFU approached limit of detection (0.48 log_10_ CFU). In this highly standardized protocol [28] rebound of infection is expected to occur in >60% of mice, a sufficiently high percentage to allow for vaccine impact evaluation. During the chemotherapy, mice were subcutaneously vaccinated or not with MVATG18598 or the empty MVATGN33.1 virus, 3 to 7 times at a dose of 10^7^ pfu/mouse/injection. Lung homogenates were plated onto agar for Mtb CFU determination 12 weeks after arrest of therapy. At the end of experiment, blood was collected from 7 randomly selected mice per group for antibody assay by ELISA and splenocytes were stimulated with Mtb Ag-specific peptides for the evaluation of T cell response by cytokine assay.

C57BL/6 mice were infected with *M*. *tuberculosis* H37Rv Pasteur (approximately 2.0 log_10_ CFU in the lungs of each mouse) using a Middlebrook Airbone Infection device (Glas-col Inc., Terre Haute, IN, USA) as described previously [29]. The experiment was done twice (n = 12 mice per group and experiment). Chemotherapy (H, 25 mg/kg; plus rifapentine (P), 10 mg/kg) was administered once a week from Week 6 to 16. Using this highly standardized protocol and antibiotic regimen, rebound of the disease is expected to occur in 100% of mice, providing room for evaluation of vaccine impact. During the chemotherapy, mice were subcutaneously vaccinated or not with MVATG18598 or the empty MVATGN33.1 virus, 3 times (Weeks 9, 12 and 15) at a dose of 10^7^ pfu/mouse/injection. Lung homogenates were plated onto agar for Mtb CFU determination 7 weeks after arrest of therapy as described previously [29] (limit of detection being 1 log_10_ CFU). At the end of experiment, mice were randomly selected for evaluation of Ag-specific T cell response (n = 8, 4 animals per experiment) by IFNγ ELISpot assay and blood was collected from all mice (n = 24, 12 animals per experiment) for antibody assay by ELISA.

For both BALB/c and C57BL/6 experiments, CFU counts in each group were first compared using a Kruskal-Wallis test. When a significant difference was obtained, then a Mann-Whitney test with Dwass, Stell, Critchlow-Fligner method for pairwise comparison of groups was applied. Percentages of relapse in each group were first compared using a Fisher Exact test. If significant *p*-value was obtained, then a Fisher Exact test for pairwise comparison between groups was applied with Benjamini-Hochberg multiplicity correction. A mouse was considered to relapse if the lung CFU count was equal to or exceeding the lower limit of detection (0.48 and 1 log_10_ CFU for BALB/c and C57BL/6 mice experiments, respectively).

### Global analysis of combined efficacy experiments

A logistic model stratified on Experiment was done with all efficacy experiments with Relapse as response and treatment group as covariate. Odds ratios and associated 95% confidence intervals were calculated.

CFU counts for all efficacy experiments were analyzed using a generalized linear model with the following effects: treatment group, experiment and the interaction between both factors. If the interaction term was not found significant, the model was re-fitted without it. If some effects were found significant, post-hoc comparisons with Tukey multiplicity adjustment were done.

## Results

### Design and *in vitro* analyses of MVATG18598

**Fig 1:**
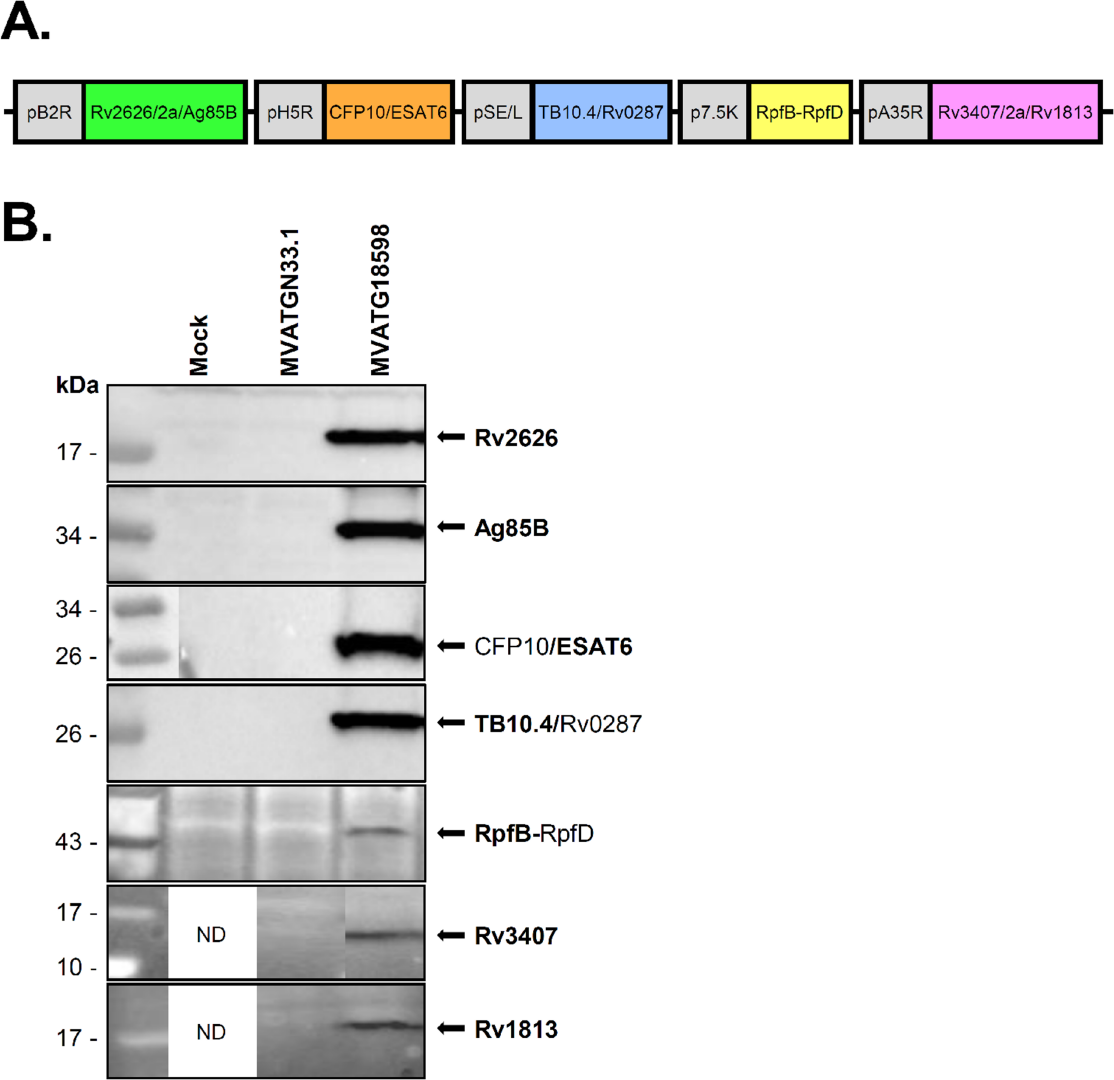
Schematic representation of antigenic expression cassettes in MVATG18598 and *in vitro* detection of antigen expression. **(A)** MVATG18598 contains the fusion Rv2626/T2a/Ag85B under the control of pB2R promoter, the fusion CFP10/ESAT6 under the control of pH5R promoter, the fusion TB10.4/Rv0287 under the control of pSE/L promoter, the fusion RpfB-RpfD under the control of p7.5K promoter and the fusion Rv3407/E2a/Rv1813 under the control of pA35R promoter. **(B)** CEF cells were infected or not (Mock) with MVATG18598 or MVATGN33.1 and cell extracts analyzed by Western blot. Fusion Rv2626/T2a/Ag85B was detected using a mouse monoclonal anti-Rv2626 antibody (expected molecular weight of cleaved product: 17.4 kDa) and a rabbit polyclonal anti-Ag85B antibody (expected molecular weight of cleaved product: 31.3 kDa). CFP10/ESAT6 fusion (expected molecular weight: 21.8 kDa) was detected using a mouse monoclonal anti-ESAT6 antibody. TB10.4/Rv0287 fusion (expected molecular weight: 21.2 kDa) was detected using an anti-TB10.4 rabbit polyclonal antibody. Fusion RpfB-RpfD (expected molecular weight: 37.0 kDa) was detected using a rabbit polyclonal anti-RpfB antibody. Fusion Rv3407/E2a/Rv1813 was detected using a rabbit polyclonal anti-Rv3407 antibody (expected molecular weight of cleaved product: 13.2 kDa) and a rabbit polyclonal anti-Rv1813 antibody (expected molecular weight of cleaved product: 12.1 kDa). ND; not done.

Expression of Mtb antigens is recognized as being a continuum during infection rather than being specifically linked to a given infectious phase [30, 31]. In our approach to Mtb vaccine development, we have worked with multiple antigens in order to cover maximum phases of the infection and have selected proteins based on several criteria such as their proven immunogenicity in animals and human populations, protection properties in animal models (when available), presence of class I and II epitopes and biochemical structure (exemplified in [19]). In the present study, we focused on the following antigens: latent phase antigens Rv2626, Rv1813 and Rv3407; active phase antigens ESAT6 (Rv3875), CFP10 (Rv3874), Rv0287, TB10.4 (Rv0288) and Ag85B (Rv1886); and resuscitation phase antigens RpfB and RpfD. The molecular configuration used to clone the 10 antigens in the MVA was the result of a selection mainly driven by capacity to generate a complex multistage vaccine fit for manufacturing development *i*.*e*. genetically stable. Antigens were combined in five separate expression-fusions each driven by a distinct promoter to generate the recombinant MVATG18598 (Fig 1A). The genetic stability of the recMVA was demonstrated by 10 successive passages in CEF. Expression of each fusion was monitored by Western blot analyses after infection of cells (Fig 1B). Strongly labeled bands detected at the expected size and corresponding to the fusions of CFP10/ESAT6 and TB10.4/Rv0287 heterodimers were detected after immune-detection with anti-ESAT6 and anti-TB10.4 antibodies. Intense bands resulting from the cleavage of T2A peptide were revealed after immune-detection with anti-Rv2626 and anti-Ag85B antibodies for the fusion Rv2626/T2a/Ag85B while faint bands resulting from the cleavage of E2A peptide were confirmed with anti-Rv3407 and anti-Rv1813 antibodies for the fusion Rv3407/E2a/Rv1813. A faint band corresponding to the expected size was detected for the fusion RpfB-RpfD.

### Immunogenicity of MVATG18598

Although IFNγ is not recognized *per se* as being a correlate of protection in TB infection, it is believed to be an important contributor to the control of mycobacterial replication [32]. ELISpot analysis was performed one week after naïve mouse immunization to investigate the specific IFNγ response induced by the MVATG18598. Immune responses specific to each expressed Mtb antigen were evaluated in two different inbred mouse strains, BALB/c (H-2^d^) and C57BL/6 (H-2^b^). Regardless of the mouse strain, a typically robust IFNγ response specific to the MVA was detected in the MVATGN33.1 control group as well as the MVATG18598-vaccinated group (>800 spots/10^6^ cells, data not shown).

**Fig 2:**
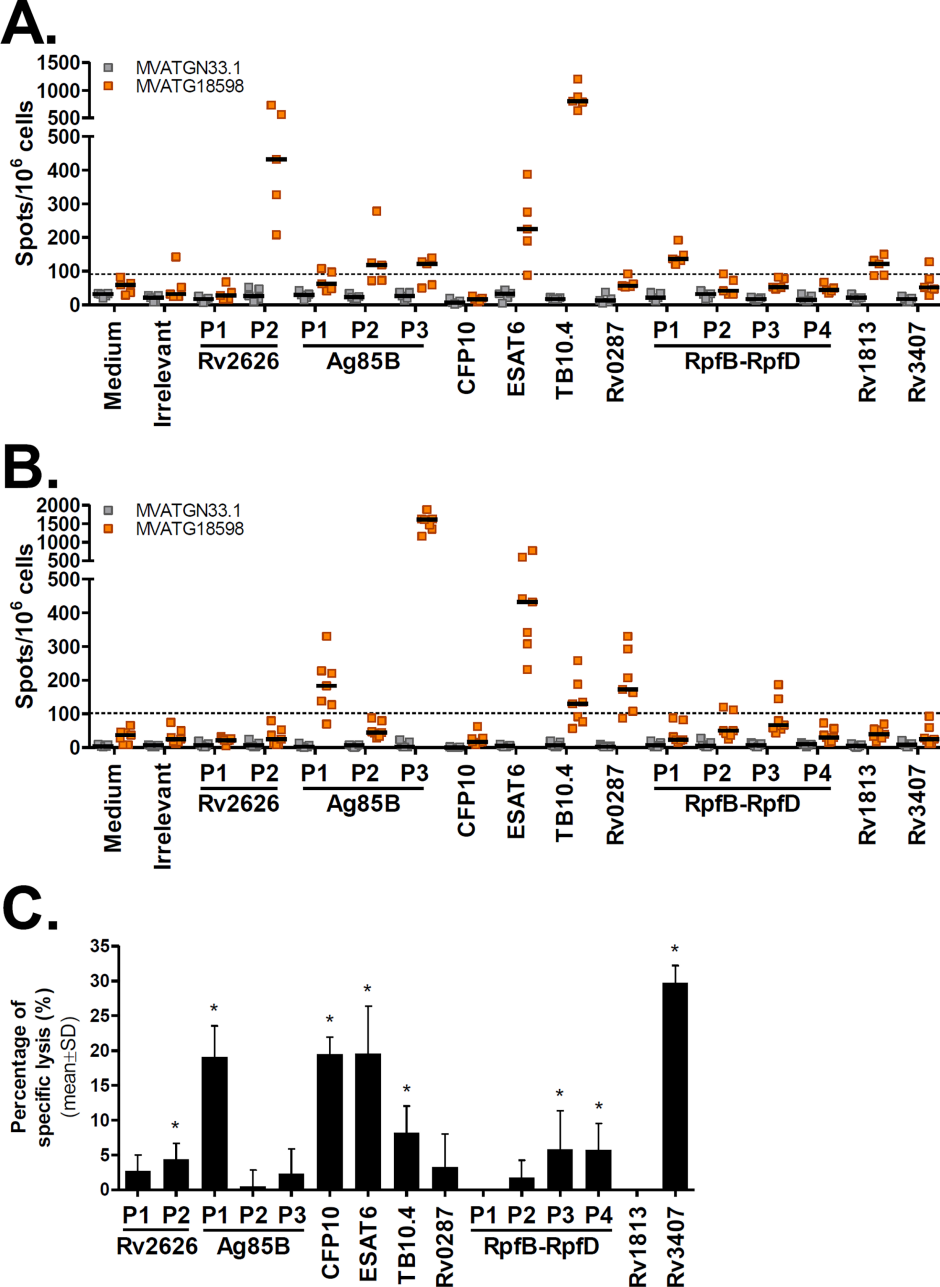
MVATG18598 immunization triggers Mtb antigen-specific cells producing IFNγ and induces cells with antigen-specific cytolytic activity. IFNγ ELISpot responses specific to Mtb antigens following injection of MVATG18598 in **(A)** BALB/c and **(B)** C57BL/6 mice. Mice were immunized once with MVATGN33.1 (grey) or MVATG18598 (orange). Results are shown as the number of IFNγ-producing cells per 10^6^ splenocytes (spots/10^6^ cells) following stimulation or not (Medium) with either an irrelevant peptide (Irrelevant) or individual pools of peptides (P) of Mtb antigens expressed by MVATG18598. Each symbol represents individual mice and black lines represent median values of each group (5 or 7 mice/group). The dotted line represents the cut-off value, above which a response was considered as positive (91 and 102 spots/10^6^ cells in (A) and (B), respectively). For each mouse strain, results are representative of independent experiments. **(C)** CTL killing response to the antigen peptide-pulsed targets in mice immunized with MVATG18598. CB6F1 mice were immunized with either MVATGN33.1 or MVATG18598. Results are expressed as mean ± S.D. of the percentage (%) of specific killing of target cells present in MVATG18598-vaccinated mice minus to those in the MVATGN33.1-vaccinated mice. For each antigen peptide pool (P), statistically significant lysis is indicated by * using a permutation resampling test. *p* value<0.05.

In BALB/c mice, Ag-specific IFNγ-producing T cells were detected for 6 out of the 10 Ag expressed by MVATG18598 (Fig 2A). The highest response was measured when cells were stimulated with TB10.4 peptides (800 spots/10^6^ cells). While robust IFNγ responses specific to ESAT6 and Rv2626 were detected (225 and 432 spots/10^6^ cells, respectively), stimulation with pools of peptides specific to Ag85B and RpfB (Pool 1 of RpfB-RpfD covering RpfB sequence) led to a readily detectable yet lower IFNγ response (ranging from 118 to 137 spots/10^6^ cells).

In C57BL/6 mice, MVATG18598 triggered IFNγ responses specific to 4 out of 10 Ag (Fig 2B). Interestingly, a very high number of cells producing IFNγ was measured in response to Ag85B stimulation (1608 spots/10^6^ cells for Pool 3). As in BALB/c mice, an ESAT6-specific IFNγ response was induced following MVATG18598 vaccination (432 spots/10^6^ cells). Lower responses were specifically detected for TB10.4 (130 spots/10^6^ cells) and, in contrast to observations in BALB/c mice, for Rv0287 (173 spots/10^6^ cells).

To assess which functional T cell subsets contribute to the MVATG18598-induced response, we investigated co-expression of the Th1 cytokines, IL2 and TNFαin addition to IFNγ, by intracellular cytokine staining (ICS) assay in vaccinated BALB/c mice. Briefly, following a single vaccination, Th1 cytokine-expressing CD4^+^ T cells were detected for TB10.4 only displaying low level of double IFNγ^+^TNFα^+^ and triple IFNγ^+^TNFα^+^IL2^+^ cell populations (S1 Fig). Similarly, TB10.4-specific CD8^+^ T cells producing IFNγ and IFNγ/TNFα were also detected post-vaccination but also for the Rv2626 antigen (similar profile). These results are in accordance with IFNγ ELISpot response since MVATG18598 triggered the highest numbers of IFNγ-producing cells in response to both TB10.4 and Rv2626 antigens in BALB/c mice (Fig 2A).

We investigated whether the T cells elicited following MVATG18598 vaccination display cytotoxicity *in vivo*. *In vivo* cytotoxic T cell (CTL) assays were performed in hybrid CB6F1 mice immunized twice with vaccine using target cells pulsed with pools of antigen-specific peptides. Mice immunized with either the MVATG18598 or the control vector generated a strong CTL killing response to the MVA peptide-pulsed targets (80–90%, data not shown). Interestingly, MVATG18598-immunized mice developed a CTL killing response specific to 8 out of 10 antigens covering the different phases of the disease and notably commonly referred to active, latency and resuscitation (Fig 2C). Different levels of CTL response were noticed. Low cytotoxic responses (<15%) were measured against target cells presenting Rv2626, TB10.4 and RpfB-RpfD antigens, and more robust killing activity (between 15 and 30%) was detected against Ag85B, CFP10, ESAT6 and Rv3407 antigen-expressing cells. Interestingly, while no IFNγ response specific to Rv3407 and CFP10 was detected in BALB/c and C57BL/6 mice, cytotoxic activities were readily measured against cells presenting these antigen peptides (29% and 20%, respectively) in hybrid CB6F1 mice.

Taken together, these results show that the multiphasic MVATG18598 vaccine is immunogenic and induces IFNγ responses in both BALB/c (6/10 Ag) and C57BL/6 (4/10 Ag) mice (S1 Table). As expected dominance of the induced responses varies with the mouse haplotype, and regardless of the mouse haplotype IFNγ-expressing cells are always detected for at least one antigen of each phase. Moreover, using three different immunological assays, the pooled data show that MVATG18598 is able to elicit immune response specific to all 10 antigens expressed by the vaccine.

### Evaluation of injection routes and positioning of MVATG18598 vaccination in a BALB/c mouse post-exposure model

**Fig 3:**
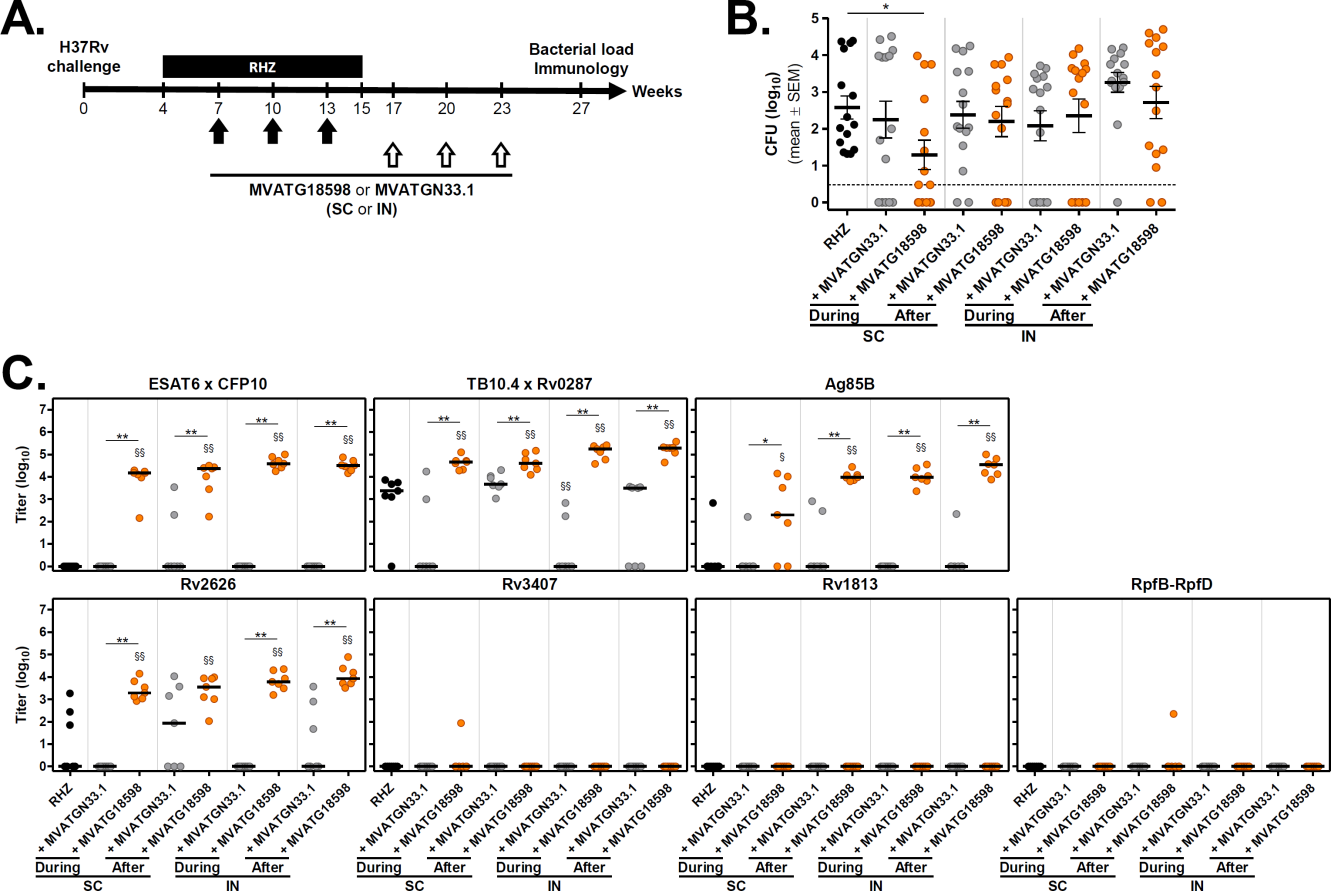
Evaluation of immunization routes and positioning of MVATG18598 in the post-exposure BALB/c mouse model. **(A)** Scheme of immunotherapy experiments. BALB/c mice were infected with a low dose aerosol of *M*. *tuberculosis* H37Rv. Four weeks later, mice were treated with an antibiotic regimen (RHZ) for 11 weeks. A subset of antibiotic-treated mice was vaccinated, through subcutaneous (SC) or intranasal (IN) routes, three times with a 3-week interval, with either MVATGN33.1 or MVATG18598 during (Weeks 7, 10 and 13) or after (Weeks 17, 20 and 23) chemotherapy. Twelve weeks after the end of the antibiotic regimen (Week 27), the number of viable bacteria in the lungs of animals was determined. **(B)** *M*. *tuberculosis* CFU counts after the completion of the antibiotic regimen in mice vaccinated or not (black) with either MVATGN33.1 (grey) or MVATG18598 (orange). Each symbol represents CFU value of individual mice. Dotted line indicates the lower limit of detection. Results are expressed as mean (black line) log_10_ CFU ± S.E.M. Statistical analysis was performed using a Mann-Whitney test. *, *p*<0.05. **(C)** Titers of antibodies specific to MVATG18598-expressed antigens at the relapse evaluation. Each symbol represents antibody titer (log_10_) of individual mice vaccinated or not (black) with either MVATGN33.1 (grey) or MVATG18598 (orange). Black line represents median value for each group. Statistical analysis was performed using a Mann-Whitney test. §, *p*<0.05 and §§, *p*<0.01 for comparison with RHZ group. *, *p*<0.05 and **, *p*<0.01 for comparison between MVATGN33.1- and MVATG18598-vaccinated groups.

Efficacy of MVATG18598 to control Mtb proliferation in infected animals was assessed in a chronic mouse post-exposure model which is widely used to evaluate novel antibiotic regimens [33]. In this model, an antibiotic regimen is administered for a sub-optimal duration such that the majority of mice (> 60%) relapse with detectable CFU 12 weeks after completing treatment. Both prevention of relapse and reduction of bacterial burden in relapsing animals are endpoints measured when testing novel therapeutic regimens. In this first proof-of-concept study, we investigated the value of MVATG18598 as an adjunct to the antibiotic regimen on these two endpoints in different vaccination settings. Systemic and mucosal delivery of MVATG18598 were tested through subcutaneous and intranasal routes of vaccination, respectively. In addition, MVATG18598 was given either during or after antibiotic therapy in order to determine the best timing of the adjunct MVA-based immunotherapy. In all experimental conditions, mice were vaccinated thrice at 3-week intervals and Mtb regrowth was assessed 12 weeks after completion of the antibiotic regimen. Global vaccination scheme is illustrated in Fig 3A.

As expected, mycobacterial regrowth occurred in a minimum of 60% of RHZ regimen-treated mice as bacilli were detected in lungs of all mice of the control group with a mean bacterial load observed at Week 27 of 2.6 ± 0.3 log_10_ CFU (Fig 3B and Table 1).

**Table 1:** Percentage of relapsing mice and bacterial burden in mice vaccinated SC or IN with MVATG18598 or empty vector, MVATGN33.1, during or after antibiotic regimen.

Treatment	Vaccination Route	Positioning *vs*. RHZ	Relapses		Bacterial load at relapse CFU (log_10_) (mean ± SEM)
			Proportion	%	
**RHZ**	**-**	**-**	15 / 15	100	2.6 ± 0.3
**RHZ + MVATGN33.1**	**SC**	**During**	10 / 15	67	2.3 ± 0.5
**RHZ + MVATG18598**			9 / 15	60	1.3 ± 0.4
**RHZ + MVATGN33.1**		**After**	13 / 15	87	2.4 ± 0.4
**RHZ + MVATG18598**			10 / 14^a^	71	2.2 ± 0.4
**RHZ + MVATGN33.1**	**IN**	**During**	10 / 15	67	2.1 ± 0.4
**RHZ + MVATG18598**			10 / 15	67	2.4 ± 0.5
**RHZ + MVATGN33.1**		**After**	14 / 15	93	3.3 ± 0.3
**RHZ + MVATG18598**			13 / 15	87	2.7 ± 0.4

R, Rifampin; H, Isoniazid; Z, Pyrazinamide.

SC, Subcutaneous; IN, Intranasal

^a^One mouse died during the experiment.

Combination of SC route and vaccination during the antibiotic treatment provided the most encouraging data. This vaccination setting resulted in a significant reduction of bacterial burden at Week 27 compared with the RHZ treated group (Fig 3B and Table 1, *p* = 0.028). A ~1.3 log_10_ CFU reduction was observed (1.3 ± 0.4 log_10_) while the empty MVATGN33.1 virus had no effect on mycobacterial regrowth. While statistical significance was not achieved (adjusted *p*-value = 0.051), the percentage of relapsing mice was reduced from 100% to 60% in the MVATG18598-vaccinated mice compared with the RHZ group in this vaccination setting. Of note, a similar trend was observed with the MVA backbone alone. None of the other tested combinations led to differences with the RHZ group (Fig 3B and Table 1), although it is interesting to note that in all groups which received MVA, a few mice systematically did not relapse.

At the end of the experiment, immune response was assessed. Antibodies recognizing individual Mtb antigens or natural heterodimers (*i*.*e*. ESAT6xCFP10 or TB10.4xRv0287) encoded by MVATG18598 were titrated in serum (Fig 3C). Antibodies specific of all antigens belonging to so-called referred active phase were detected. High levels of antibodies capable to bind the ESAT6xCFP10 complex were significantly measured in all groups of mice receiving MVATG18598 (median titers between 4 and 5 log_10_), whereas no antibodies were detected in mice treated with antibiotics only or receiving the empty MVATGN33.1. A similar pattern of antibody response was also observed for Ag85B antigen. Although TB10.4xRv0287-specific antibodies were present in serum of antibiotics only-treated mice or animals injected with the control MVATGN33.1 after antibiotic therapy (3–4 log_10_), higher amounts of antibodies were induced in mice vaccinated with MVATG18598 (4–6 log_10_) regardless of the positioning and route of vaccination. Among the described latent antigens, Rv2626 is the only antigen for which antibody response was measured, higher levels of antibodies being detected in MVATG18598 vaccinated mice, regardless of kinetics and routes of vaccination. No antibodies directed against RpfB-RpfD were detected. Overall, MVATG18598 vaccination led to a sustained significant higher level of antibodies which were poorly or not induced in control mice. Injection route or vaccine positioning did not impact antibody detection. No correlation between CFU counts and antibody levels was demonstrated using Spearman correlation test (data not shown).

### Evaluation of MVATG18598 vaccination schedule in a BALB/c mouse post-exposure model

**Fig 4:**
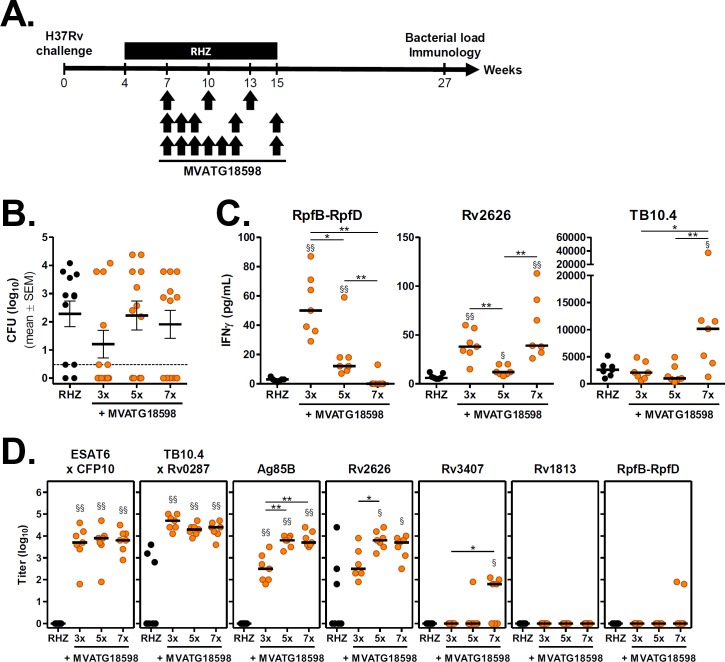
Efficacy of MVATG18598 vaccination schedule in the post-exposure BALB/c mouse model. **(A)** Scheme of immunotherapy experiments. BALB/c mice were infected and treated with an antibiotic regimen (RHZ) as described previously. Three subsets of antibiotic-treated mice received 3 (Weeks 7, 10 and 13), 5 (Weeks 7, 8, 9, 12, 15) or 7 (Weeks 7, 8, 9, 10, 11, 12 and 15) injections of MVATG18598 (10^7^ pfu/injection) through SC route during chemotherapy. Twelve weeks after the end of antibiotic regimen (Week 27), the number of viable bacteria in the lungs of animals was determined and Ag-specific immune responses were assayed. **(B)** *M*. *tuberculosis* CFU counts in mice after the completion of antibiotic regimen alone (black) or in combination with MVATG18598 injected 3, 5 or 7 times (orange). Each symbol represents CFU value of individual mice. Dotted line indicates the lower limit of detection. Results are expressed as mean (black line) log_10_ CFU ± S.E.M. **(C)** IFNγ responses following stimulation of splenocytes from unvaccinated (black) or MVATG18598-vaccinated (orange) antibiotic-treated BALB/c mice with Mtb antigen-specific peptides. Cells were stimulated or not with pools of peptides covering the full sequence of RpfB-RpfD, Rv2626 or TB10.4 as described in Material and Methods section. IFNγ production was then determined in supernatants. Experimental group consisted of 7 mice per group randomly selected at the end of the experiment. Results are expressed as concentration of IFNγ (pg/mL). Symbols represent individual mice and black lines represent median value of each group. For each mouse, the basal level corresponding to the unstimulated experimental condition was subtracted. Statistical analysis was performed using a Mann-Whitney test: *, *p*<0.05 and **, *p*<0.01 for comparison between MVATG18598 groups; §, *p*<0.05 and §§, *p*<0.01 for comparison with RHZ group. **(D)** Titers of antibodies specific to MVATG18598-expressed antigens at the relapse evaluation. Each symbol represents antibody titer (log_10_) of individual mice vaccinated or not (black) with MVATG18598 (orange). Black line represents median value for each group. Statistical analysis was performed using a Mann-Whitney test. §, *p*<0.05 and §§, *p*<0.01 for comparison with RHZ group. *, *p*<0.05 and **, *p*<0.01 for comparison between MVATG18598-vaccinated groups.

Additional experiments were undertaken in the same post-exposure model, to evaluate impact of number of injections on therapeutic endpoints described above. For these experiments, the optimal vaccination setting identified in prior evaluation *i*.*e*. SC route and vaccination in presence of antibiotics, was kept. We have successfully used closely spaced and repetitive schedules of administration with MVA-based therapeutic vaccines in both clinical phase 2 developments in oncology and chronic infectious diseases (TG4010 and TG4040 respectively, [34, 35]). Those guided our interest in testing 3, 5 or 7 SC injections of MVATG18598 starting 3 weeks after start of chemotherapy (Fig 4A).

Bacterial loads in the lungs were assessed 12 weeks after end of antibiotic therapy. In the control group, the disease rebounded in 83% of RHZ-treated mice with a mean CFU value of 2.3 ± 0.5 log_10_, according to expectations (Fig 4B and Table 2).

**Table 2:** Percentage of relapsing mice and bacterial burden in mice vaccinated SC with increasing vaccination number of MVATG18598 during antibiotic regimen.

Treatment	No. of inject.	Relapses		Bacterial load at relapse CFU (log_10_) (mean ± SEM)
		Proportion	%	
**RHZ**	**-**	10 / 12	83	2.3 ± 0.5
**RHZ + MVATG18598**	**3x**	6 / 12	50	1.2 ± 0.5
**RHZ + MVATG18598**	**5x**	8 / 12	67	2.2 ± 0.5
**RHZ + MVATG18598**	**7x**	7 / 12	58	1.9 ± 0.5

R, Rifampin; H, Isoniazid; Z, Pyrazinamide.

While bacterial loads in groups of mice vaccinated 5 and 7 times with MVATG18598 were similar to the RHZ group (2.2 ± 0.5 and 1.9 ± 0.5 log_10_, respectively), the lowest CFU count was measured when mice received the 3 injections regimen (1.2 ± 0.5 log_10_). Interestingly, the mean bacterial load difference with the RHZ control group was 1.1 log_10_, confirming the reduction level observed in the previous experiment under the same vaccination settings (Fig 3B and Table 1). Similarly, the lowest number of relapses was observed when the MVATG18598 was delivered 3 times. In this group, Mtb was not detected in half of mice (Fig 4B and Table 2), a protection level similar to that from the previous study. MVATGN33.1 was not administered in the protocol.

We investigated immune responses specific to a representative subgroup of antigens expressed by MVATG18598 at the end of the experiment, *i*.*e*. 12 or 14 weeks after the last vaccination. We focused on RpfB-RpfD, Rv2626 and TB10.4 which are described in the literature as representatives of the 3 phases of the disease (resuscitation, latency and active disease, respectively) and display good immunogenicity in naïve BALB/c mice (S1 Table). Spleen cells of 7 mice per group randomly selected the day of euthanasia were stimulated *ex vivo* with peptide pools spanning the 3 antigens. Production of IFNγ in the supernatant was then measured as described in the Material and Methods section. As shown in Fig 4C, although low levels of IFNγ were detected in response to RpfB-RpfD and Rv2626 peptides, significant higher amounts of IFNγ were measured in mice vaccinated with MVATG18598 compared with RHZ-treated mice only (near undetectable levels). Higher production of IFNγ was measured following stimulation with TB10.4 peptides in particular in the group which received 7 vaccinations (10 155 pg/mL as compared with 2 572 pg/mL in RHZ group). Intensity of the IFNγ response detected in this assay appeared to depend on both the antigen tested and the schedule of administration.

Besides T cell responses, antibody responses specific to MVATG18598-expressed antigens were investigated in serum of the same 7 mice. As in the previous efficacy protocol, all antigens belonging to the active phase of the infection triggered specific antibody response (Fig 4D). High antibody level specific to ESAT6xCFP10 complex was measured in mice vaccinated with MVATG18598 (3.7 log_10_) in comparison with RHZ only-treated mice. No difference was noticed between the three immunization schedules. In this model, majority of antibiotics only-treated mice did not display TB10.4xRv0287-specific antibodies, and strong levels of antibodies was measured in the three MVATG18598-vaccinated groups. For both Ag85B and Rv2626, robust antibody-specific responses were observed in MVATG18598-vaccinated mice with a significant lower level of antibody noticed in mice vaccinated three times. For Rv3407, as in the previous protocol no response was detected in the 3x vaccinations schedule, but a signal was seen when MVATG18598 was injected 7 times. No response for Rv1813 and RpfB-RpfD antigens was measured for any vaccination schedule.

Results of this experiment together with those of the first study strongly suggest that the 3x injection schedule by the SC route of the MVATG18598 in combination with antibiotics results in the most important impact in the BALB/c post-exposure model. Moreover, vaccination resulted in both sustained Ag-specific antibody and Th1-type cell responses in Mtb-infected mice that were still detected 14 weeks after last immunization. These responses were not linked to the lung bacterial burden (Spearman correlation test, data not shown). Although it is not possible at that stage to conclude on the specific role of the detected immune responses in the therapeutic impact observed, clearly those are not seen in the antibiotic-treated group only.

### Evaluation of MVATG18598 therapeutic efficacy in a C57BL/6 mouse post-exposure model

**Fig 5:**
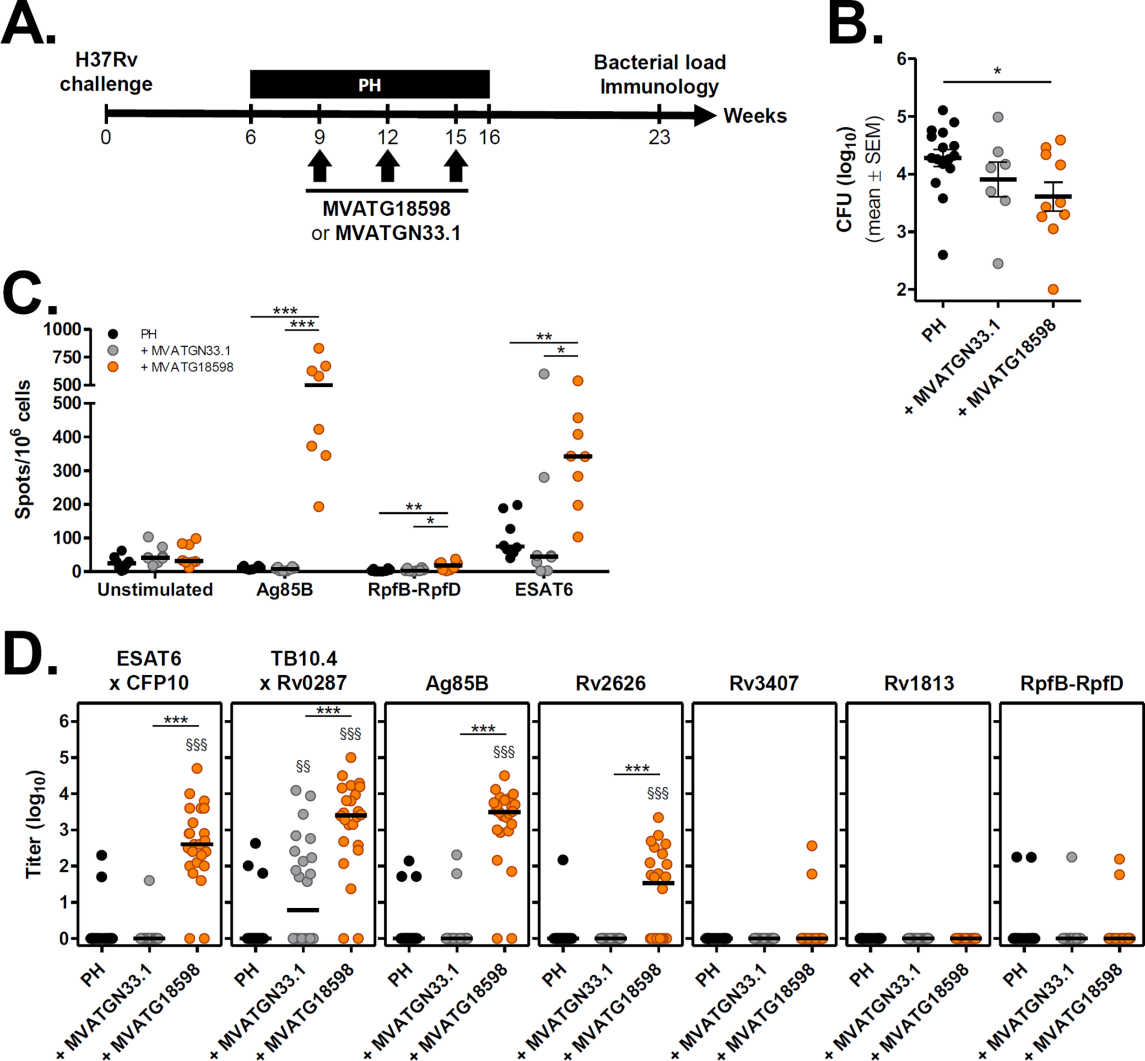
Efficacy of MVATG18598 in the post-exposure C57BL/6 mouse model. **(A)** Scheme of immunotherapy experiments. C57BL/6 mice were infected and treated with an antibiotic regimen (P, rifapentine and H, isoniazid) as described in Material and Methods section. Two subsets of antibiotic-treated mice received 3 injections of either MVATGN33.1 or MVATG18598 through SC route during chemotherapy (Weeks 9, 12 and 15). Seven weeks after end of antibiotic regimen (Week 23), number of viable bacteria in the lungs of animals was determined and Ag-specific immune responses were assayed. **(B)** *M*. *tuberculosis* CFU counts in the lungs after the completion of the antibiotic regimen alone (PH, black) or in combination with MVATGN33.1 (grey) or MVATG18598 (orange). Each symbol represents the CFU value of an individual mouse. Results are expressed as mean (black line) log_10_ CFU ± S.E.M. Statistical analysis was performed using a Mann-Whitney test. *, *p*<0.05. **(C)** IFNγ responses following stimulation of splenocytes from MVATGN33.1- or MVATG18598-vaccinated C57BL/6 mice with Mtb antigen-specific peptides. Cells were stimulated or not with pool of peptides specific to the Ag85B (Pool 3), RpfB-RpfD (Pool 3) or ESAT6 for IFNγ ELISpot assay as described in Material and Methods section. Results are shown as the number of IFNγ-producing cells per 10^6^ splenocytes (spots/10^6^ cells) following stimulation or not (Unstimulated) with pools of peptides. Each experimental group is composed of 8 mice resulting from two cumulated runs with 4 mice per run randomly selected at the end of the *in vivo* experiment part. Each symbol represents an individual mouse and black lines represent median values of each group. Statistical analysis was performed using a Mann-Whitney test: *, *p*<0.05; **, *p*<0.01 and ***, *p*<0.001. **(D)** Titers of antibodies specific to MVATG18598-expressed antigens at the relapse evaluation. Each symbol represents antibody titer (log_10_) of individual mice vaccinated or not (black) with either MVATGN33.1 (grey) or MVATG18598 (orange). Black line represents median value for each group. Statistical analysis was performed using a Mann-Whitney test. §§, *p*<0.01 and §§§, *p*<0.001 for comparison with RHZ group. ***, *p*<0.001 for comparison between MVATGN33.1- and MVATG18598-vaccinated groups.

The post-exposure chronic infection model established in C57BL/6 mice, similar in its concept to the BALB/c model, has already been described for evaluating therapeutic candidate vaccines [11, 29]. It differs from the BALB/c model in the composition of antibiotic regimen applied and its schedule of administration, the percentage of relapsing mice post-antibiotic arrest (which is routinely 100%) and the duration of post-treatment follow-up. Above all, being a different mouse H2 haplotype, it represents an opportunity to validate cross-capacity of vaccine-induced immune responses to impact treatment outcomes in a different immunological background and thus better mimics the clinical situation of vaccinees expected to harbor a variety of HLA types. We evaluated MVATG18598 therapeutic activity in this model using the optimal route and schedule of administration identified previously. Global protocol scheme is outlined in Fig 5A. Mice were infected with a low dose aerosol and treated with an isoniazid and rifapentine regimen from Week 6 to 16 post-infection. MVATG18598 was given through subcutaneous route 3 times at 3-week intervals starting at Week 9 post-infection. As a negative control, a subset of infected mice was vaccinated with the empty MVATGN33.1 virus. Bacterial burden in vaccinated mice was assessed 7 weeks after therapy completion (Week 23) and compared to the control group treated with antibiotics only.

Two independent experiments using 12 mice per group were run. Unexpectedly, technical issues were encountered during the *in vitro* mycobacterial culture phase resulting in incomplete CFU data for some animals. Only results of exploitable individual mice data from the two pooled experiments were used for statistical comparison between groups. A total of 16, 7 and 10 mice for the PH, MVATGN33.1 and MVATG18598 groups, respectively, were counted (Fig 5B and Table 3).

**Table 3:** Percentage of relapsing mice and bacterial burden in mice vaccinated SC with MVATG18598 during antibiotic regimen.

Treatment	Relapses		Bacterial load at relapse CFU (log_10_) (mean ± SEM)
	Proportion	%	
**PH**	16 / 16	100	4.3 ± 0.1
**PH + MVATGN33.1**	7 / 7	100	3.9 ± 0.3
**PH + MVATG18598**	10 / 10	100	3.6 ± 0.3

P, Rifapentine; H, Isoniazid.

All mice relapsed, as expected. Lung CFU counts reached the expected level in the antibiotic control group (4.3 ± 0.1 log_10_). While vaccination with MVATGN33.1 did not impact bacterial burden, a statistically significant reduction in CFU count was measured in mice vaccinated with MVATG18598 (0.7 ± 0.3 log_10_ reduction, Fig 5B and Table 3). This result is in agreement with the impact of MVATG18598 vaccination on CFU counts in BALB/c mice.

We monitored specific immune responses in vaccinated C57BL/6 mice at the end of each independently-run experiment. As it was not technically possible to test T cell response against the 10 antigens, we focused on 3 antigens which display either strong immunogenicity in naïve C57BL/6 mice (Ag85B and ESAT6) or are novel in the vaccine field (RpfB-RpfD) (S1 Table). As seen with BALB/c mice, the intensity of detected responses differed with the treatment regimen and the antigen tested. The most robust responses were seen in the MVATG18598-vaccinated group and directed at Ag85B and ESAT6. The two other groups (‘PH’ and ‘+ MVATGN33.1’) yielded little or no response specific to these 2 antigens (Fig 5C). In the ‘+ MVATG18598’ group, significant high numbers of Ag85B- and ESAT6-specific cells producing IFNγ ranged from 502 and 343 spots/10^6^ cells, respectively. Very low level of cells specific to the RpfB-RpfD antigen was observed (18 spots/10^6^ cells).

Antibody responses directed against MVATG18598-expressed antigens were also investigated by ELISA at the end of each experiment. Positive signals were detected for the same antigens as in BALB/c experiments (Fig 5D). All antigens of the active phase were recognized by antibodies present in sera of mice vaccinated with MVATG18598. While no antibodies specific to ESAT6xCFP10 complex or Ag85B were detected in the control groups mice, higher levels were observed in mice vaccinated with MVATG18598 (2.6 and 3.5 log_10_, respectively). High levels of anti-TB10.4xRv0287 antibodies were measured in mice administered with MVATG18598 (22/24 mice, 3.4 log_10_) while antibodies could barely be detected in antibiotics only-treated mice (3/24 mice). Unexpectedly significant antibodies were measured in mice vaccinated with the empty MVATGN33.1 (12/24 mice, 0.8 log_10_) suggesting that the MVA virus might have boosted specific antibody response through innate immunity. Of note, Rv2626-specific antibodies were significantly measured in MVATG18598-vaccinated C57BL/6 mice (1.5 log_10_) in comparison with control groups. No antibodies specific of Rv3407, Rv1813 and RpfB-RpfD was detected except sporadically. Although high antigen-specific T and B cell responses were mounted in vaccinated mice, no correlation could be found with the lung bacterial burden (data not shown).

Overall, the protective efficacy of MVATG18598 against Mtb regrowth (approx. 1 log_10_ CFU mean reduction) was confirmed using two post-exposure mouse models. In both models, MVATG18598 vaccination triggered a sustained Ag-specific memory response (T and B cells) directed at multiple antigens.

### Pooled analysis of therapeutic protocols performed with MVATG18598 in two Mtb post-exposure mouse models

To strengthen our analysis and interpretation, we pooled data from the three comparable experiments in which MVATG18598 was subcutaneously administered 3 times at 3-week intervals during antibiotic treatment. Tables 4 and 5 summarize these data and outline the statistical tools used for the analyses.

**Table 4:** Statistical analyses of reactivation of pooled post-exposure experiments performed in both BALB/c and C57BL/6 mouse models. Groups of mice co-treated with antibiotics (ATB) and MVATG18598 or MVATGN33.1 given thrice subcutaneously during antibiotic therapy were analyzed.

Reactivation		
Group comparison	*p*-value	Odds Ratio [95% CI]
‘ATB’ vs. ‘ATB+MVATGN33.1’	0.051	9.2 [1.3;65.4]
‘ATB’ vs. ‘ATB+MVATG18598’	0.007	9.9 [1.9;50.2]
‘ATB+MVATGN33.1’ vs. ‘ATB+MVATG18598’	0.167	1.1 [0.3;4.6]

Stratified logistic regression test, *p*-value = 0.020

**Table 5:** Statistical analyses of bacterial load of pooled post-exposure experiments performed in both BALB/c and C57BL/6 mouse models. Groups of mice co-treated with antibiotics (ATB) and MVATG18598 or MVATGN33.1 given thrice subcutaneously during antibiotic therapy were analyzed.

Bacterial load		
Group comparison	Adjusted *p*-value	Estimate CFU difference ± S.E (log_10_)
‘ATB’ vs. ‘ATB+MVATGN33.1’	0.678	0.32 ± 0.37
‘ATB’ vs. ‘ATB+MVATG18598’	0.003	1.04 ± 0.31
‘ATB+MVATGN33.1’ vs. ‘ATB+MVATG18598’	0.151	0.72 ± 0.39

Generalized Linear Model test, *p*-value for Group = 0.004

A statistical difference was seen between the MVATG18598-vaccinated group and the antibiotic-only treated group in favor of addition of the MVATG18598 to the antibiotic regimen. On impact on relapse, a significant effect of group was found (*p*-value = 0.020) in stratified logistic regression: the only significant parameter was for ‘ATB’ *vs*. ‘ATB+MVATG18598’ with a *p*-value of 0.007 and mice receiving ATB only were found to be approximately 10 times more likely to relapse (Table 4, odds ratio: 9.9 with 95% confidence interval [1.9; 50.2]). On impact of lung CFU load, the interaction term was not found to be significant but a significant impact of experiment (not shown) and group (*p*-value = 0.004) were found: pairwise comparison showed a significantly lower mean CFU count for ‘ATB+MVATG18598’ *vs*. ‘ATB’ (Table 5, adjusted *p*-value was 0.003) with a mean estimated CFU difference of 1.04 log_10_. This analysis illustrates the dual activity of the MVATG18598 vaccine in the context of 3 injections and when used as adjunct to antibiotic treatment *i*.*e*. capacity to prevent relapse and to reduce bacilli load in lungs of mice.

## Discussion

The goal of immunotherapies is to modulate the diseased host immune system in a positive way via the triggering of immune responses capable to contribute to the control or elimination of the disease/infection. Both specific (therapeutic vaccines) and non-specific (host-directed therapies) products are today actively developed in the field of oncology and chronic infectious diseases [36]. Immunotherapeutics are typically developed in combination with standards-of-care (SOC), as adjunct therapy, rather than in stand-alone scenarios. The supportive concept is to bring-in participation of the host immune system which is either not (*e*.*g*. in case of direct acting-antivirals) or insufficiently (*e*.*g*. in case of chemotherapies) stimulated by the SOC. In the case of tuberculosis, increasing efforts are deployed to develop immunotherapeutics in particular with regard to the fight against multidrug-resistant tuberculosis [37–39]. We report here the first multistage viral vector-based therapeutic vaccine tested in the tuberculosis field and describe its value in increasing the efficacy of chemotherapy regimens in two murine post-exposure models.

Sophisticated molecular engineering (use of multiple promoters, self-cleaving peptides, co-expression of naturally partnered proteins) enabled the successful generation of a recombinant MVA, MVATG18598, expressing 10 Mtb antigens spanning different phases of infection. This vaccine is unique in the field of TB recombinant vaccines which typically encompass 3–5 Mtb antigens [9], but also in MVA-based vaccination at large [17]. A key feature of this MVA is its genetic stability qualifying it for manufacturing and further clinical development. We show in naïve mice that MVATG18598 is immunogenic in particular in its ability to generate IFNγ-producing T cells, a cytokine known to be essential to control Mtb infection [32]. It is also able to trigger specific cytotoxic T lymphocytes (CTL), a feature rarely described in the TB vaccine field. The role of T cell-driven cytotoxic activity has been described in earlier experimental studies and shown to be required for optimal immunity against TB [40, 41], although direct human confirmatory data are still lacking. Polyfunctionality of the MVA educated T cells in naïve mice was quite limited in the experimental settings applied (triple cytokines CD4 T cell producers detected for only one antigen). Testing multiple MVA injections in naïve mice, both BALB/c and C57BL/6, as in efficacy protocols would deserve to be explored to investigate whether MVATG18598 induces further Ag-specific polyfunctional T cells. Unfortunately, we could not document it in the infected mouse models. MVATG18598-induced immune responses target multiple antigens, with variable intensity but systematically including at least one antigen representative of distinct phases of infection. One concept behind the design of our vaccine was to include sufficient number of antigens so as to ensure a maximum immune coverage (all phases of infection) over multiple HLA types. The present data, combined with earlier demonstration that we performed with another highly complexed recMVA (14 antigens) tested both in mice and primates [19], illustrate the capacity of such complex MVAs to induce broad immune responses covering different haplotypes.

There are many elements to take into consideration when evaluating a candidate therapeutic vaccine. Besides considerations related to the route of administration, dose and schedule, the time of vaccine introduction with respect to the antibiotic treatment is a critical factor. We performed multiple protocols in two different mouse models to address these different elements. Optimal configuration for MVATG18598 activity was identified as relying on the SC route of administration concomitantly with the antibiotic regimen as well as on the application of a rather limited number of doses (3 rather than 5 or 7). The resulting impact of adding MVATG18598 to antibiotic regimens was seen at two levels: impact on prevention of relapse following treatment arrest as well as on lung bacilli load in long-term follow-ups. Effects were also seen (mainly on prevention of relapse) with the empty MVA vector, likely due MVA-capacity to trigger innate immunity (discussed below). Prevention of relapse, seen in the BALB/c model, is a particularly important endpoint. The sterilizing effect observed in a model based on transition to chronic infection supports the notion that it is possible to manipulate the infected host immune system to contribute to bacillary clearance [10, 42]. The BALB/c model used here is the only one to our knowledge to have shown robust correlation between efficacy obtained with new drug regimens (mainly on prevention of relapse) and results seen in the clinic. For instance, novel bedaquiline-based regimens have been successfully tested in this mouse model and clinical data generated subsequently in MDR- and XDR-TB infected patients confirmed the value of this novel antibiotic in patients [43, 44]. Further studies are required to identify parameters and pathways triggered by MVATG18598 that are susceptible to play a role in restoration of expected defective Th1-reponse (*e*.*g*. level of expressed antigens, phenotype of T-cells, localization, etc.).

We were not able to perform longitudinal analysis of Mtb specific responses over the course of our experiments but observed in the BALB/c model that the IFNγresponse tested at the end of study (for the 3x-injection schedule, 14 weeks post-vaccination) was specific to 2 of 3 vaccine antigens evaluated (RpfB-RpfD and Rv2626) and only seen in the vaccinated group (Fig 4C). Response specific to the third evaluated vaccine antigen (TB10.4) was strongly boosted compared with the RHZ treated group (Fig 4C). We observed similar results in C57BL/6 mice in which Ag85B-, RpfB-RpfD- and ESAT6-specific responses were basically only detected in the vaccine arm. Effect of the MVATG18598 on Mtb antigen-specific immunogenicity was further illustrated on B-cell response. Antibodies recognizing all active antigens and the latent Rv2626 protein expressed by the vaccine were still detected 14 weeks after the last immunization. While we acknowledge our limitation in analyzing induced T-cell responses (only a limited set of assays targeting a few selected antigens were used here), these observations point to a contribution of vaccine-induced immunity on the protective effects observed. Clearly additional immune analyses are needed to clarify the role played by the specific immunity detected in the vaccinee groups and the protection observed in particular that directed at latency and resuscitation antigens which have not yet been tested in TB vaccine development. The separate contribution of the 10 vaccine-encoded antigens and/or that of their combination in the observed sterilizing immunity also requires further exploration.

Other therapeutic vaccines aiming at improving SOC have been described. These vaccines, mostly tested in mouse models of either chronic infections (such as used in our study [11, 13, 29], or in lethal/ lesion-prone models [12] have been shown to impact bacterial load post-chemotherapy arrest with an efficacy which appears similar to that observed with MVATG18598 (approx. 1 log_10_ CFU lung reduction). No significant impact on relapse has been reported in the various published mouse data with these vaccines. The vaccines were mostly given after stopping antibiotic treatment. One such vaccine (ID93/GLA-SE) was brought to a non-human primate therapeutic model [12] in which it was shown to prevent lung pathology and for 2 of 5 vaccinees, led to infection resolution. Another candidate (AEC/BC02) was tested in guinea pigs [45] and reported to improve lung pathology as well as decrease lung CFU loads but with no reported effect on relapse or resolution of infection. The encouraging results seen in the lethal NHP model with the ID93/GLA-SE vaccine which has been designed and shown to induce a T-cell based immunity, are in strong support of the need to enhance Th1 oriented responses in order to achieve sterilization of infection and control of lung necrosis/inflammation in a post-antibiotic positioning [12]. The ID93/GLA-SE vaccine includes 4 adjuvanted antigens counting 3 virulence factors (Rv2608, Rv3619, Rv3620) and one latency antigen (Rv1813). MVATG18598 expresses the same latency antigen in addition to two others, Rv2626 and Rv3407 while the H56 vaccine, which showed protection in the C57BL/6 murine chronic post-exposure model, includes yet a different latency protein (Rv2660c, [11]). Interestingly, 2 of 3 of MVATG18598-expressed latency antigens (Rv2626 and Rv3407) displayed a CTL cytotoxic activity beyond being producers of IFNγ. It would be of interest to dissect the role of immune responses specific to latency antigens in all recombinant therapeutic vaccines developed today containing this class of antigen as stimulating an immune response targeting dormant, metabolically inactive and non-replicating bacteria would be an important endpoint in improvement of MDR management [42, 46].

The contribution of innate immunity in therapeutic effects observed with the various developed vaccines has not yet been addressed. In most published studies, either the adjuvant alone is not systematically tested [12], or it is in place of a mock treatment [11] or when all controls are included, adjuvants alone appear to have some effect [45]. In our study, the MVA backbone (MVATGN33.1) was tested and revealed some efficacy in the first experiment in BALB/c mice (Fig 3B). MVA is known to be a very strong inducer of innate immunity including stimulation of Type I IFN pathways (TLR2/6, MDR-5), the inflammasome (AIM2/NALP3), and NK cells [47–49]. Those pathways should be further studied to clarify their role in the protective effect observed linked to the MVA backbone. In a recent prophylactic study, Beverley *et al*. evaluated an Ag85A-expressing murine CMV vaccine and found that activation of NK cells by the MCMV vector itself provided early non-specific protection against *M*. *tuberculosis* [50]. Global protection was enhanced when the Ag85A-specific response was induced by the recMCMV vaccine. Increasing evidence is being collected supporting an important role played by innate immune responses in both natural and vaccine-induced protection against Mtb infection. In particular the role of NK cells in immune-based therapeutic protection needs to be studied and would, if established, provide an interesting advantage to MVA-based vaccines. Finally, an interesting observation made in the field so far is that all evaluated therapeutic vaccines, whether in mice, guinea pigs or NHP, display a good safety profile. Even in our experiment in which we tested up to 7 injections of the MVATG18598, we did not observe any major side effects in spite of induction of strong, long lasting memory responses (data not shown).

There are different advantages of the MVATG18598 compared with other developed therapeutic vaccines. The well-established clinical safety profile of the MVA platform [51] and early observations made here on lack of side effects/exacerbation of Mtb infection when administered multiple times as adjunct therapy, constitute a strong basis for clinical administration in a TB-therapeutic context. The 10-antigen-based composition and lack of major immune-dominance increase the odds to mount specific responses against representatives of different infection phases independent of the vaccinees’ HLA type. An important observation in our study is that we did observe efficacy when the MVA vaccine was concomitantly administered with the antibiotic regimen rather than post-treatment. Clearly our principal aim will ultimately be to shorten the course of therapy in particular in MDR patients. With improvement of drug regimens recently reported *i*.*e*. shortening to 9 months versus the classical 18/24 months of treatment in this population, it will be critical to administer the vaccine early enough after initiation of therapy to ensure a rapid impact in order to bring a novel treatment paradigm to the patients.

Overall, kinetics of therapeutic vaccine administration, such as for MVATG18598, should be carefully studied in clinical settings together with appropriate safety and efficacy measurements and should include biomarker/signature analysis. Pursuing further animal studies represents a challenge and will either require development of novel models using the same antibiotic regimens as used in the MDR-clinic today and /or tomorrow (expected novel regimens) in order to best mimic clinical trial development. Such models will yet likely only capture part of the vaccine-associated mechanism of action (*e*.*g*. innate immunity can differ widely between species) hampering proper interpretation and transposition of results from animals to humans. Obviously, these considerations are very different from the small-molecule, antibiotic development field which does not rely on immune-mechanisms. With all these considerations in mind, the proof-of-activity seen with the MVATG18598, in particular with respect to relapse testing, is very encouraging. It is possible that combining routes of injections which has shown to yield optimal immune responses [52], together with a multi-site injection approach recently used with a vectored cancer vaccine [53] may still result in enhanced efficacy of the MVATG18598.

In conclusion, we have developed an original multistage recombinant MVA-TB therapeutic vaccine which has shown activity in post-exposure preclinical models and is fit for manufacturing development. Its value in clinical settings, most specifically to contribute to treatment of difficult to treat patients such as MDR- and XDR-TB patients, awaits further development.

## Supporting information

S1 Protocol Peptide library and triple ICS assay. (DOCX)

S1 Fig CD4 and CD8 T cell responses induced by MVATG18598 monitored by an intracellular IFNγ, TNFα and IL2 staining assay in naive BALB/c mice. Mice were immunized once with MVATG18598 or the empty vector MVATGN33.1, as a negative control. Cells were stimulated with Mtb peptide pools, the MVA vector-specific VGP peptide or an irrelevant E7 peptide. Results are presented as the percentage of single, double and triple positive CD8 or CD4 T cells for IFNγ, TNFα and IL2 among total CD4 and CD8 T cell populations, respectively. Each symbol represents response from individual mice and line represents median response. Cut-off values are indicated as dotted lines. Only cytokine-producing cell population above the cut-off values are represented. For each cell population, background signal obtained in unstimulated cells condition was subtracted. No cytokine-producing cell population was detected in mice vaccinated with MVATGN33.1 or following stimulation with the irrelevant E7 protein (not shown). Multi-cytokine-producing CD4 and CD8 T cells specific to the MVA vector were also detected in the MVATGN33.1-vaccinated control group as well as the MVATG18598-vaccinated group (data not shown).
(PPTX)

S1 Table Summary of immune responses induced by MVATG18598 vaccine in naive BALB/c, C57BL/6 and CB6F1 mice. Resuscit., Resuscitation. IFNγ ELISpot: group median value of spots detected using the following ranking: -, median < cut-off; +, 1x cut-off < median < 2x cut-off; ++, 2x cut-off < median < 3x cut-off; +++, 3x < median < 5x; ++++, median > 5x cut-off. CTL: -, no activity; +, CTL < 20%; ++, 20% < CTL < 30%).
(PPTX)
