# Supplementary material for: A multi-antigenic MVA vaccine increases efficacy of combination chemotherapy against Mycobacterium tuberculosis
Source: PLoS One. 2018 May 2;13(5):e0196815. doi: 10.1371/journal.pone.0196815 (PMC5931632; doi:10.1371/journal.pone.0196815)
Supplement: S1 Protocol — (DOCX) [file pone.0196815.s001.docx]

**S1 Protocol**

**Peptide library**

In order to stimulate *ex vivo* splenocytes from immunized mice, we used a peptide library (synthesized by ProImmune using protocol prospector LCMS) constituted by 15-mer peptides overlapping by 11 aa. For each antigen, pools of peptides were constituted, each pool containing 25 peptides maximum. Therefore, 1 to 4 pools for each respective antigen were needed in order to cover the full length of the given antigens as described below:

- RpfB-RpfD was covered by 4 pools of 22 peptides for the first 3 pools and 19 peptides for the fourth pool. Pool 1: 22 peptides covering RpfB residues 30 to 127; Pool 2: 22 peptides covering RpfB residues 117 to 215; Pool 3: 22 peptides covering RpfB residues 205 to 284 and RpfD residues 53 to 71; Pool 4: 19 peptides covering RpfD residues 61 to 146.
- Rv1813 was covered by 1 pool of 25 peptides. Pool 1: 18 peptides covering Rv1813 residues 34 to 143.
- Rv3407 was covered by 1 pool of 22 peptides. Pool 1: 18 peptides covering Rv3407 residues 1 to 99.
- Rv2626 was covered by 2 pools of 17 and 16 peptides. Pool 1: 17 peptides covering Rv2626 residues 1 to 79; Pool 2: 16 peptides covering Rv2626 residues 69 to 143.
- Ag85B was covered by 3 pools of 23 peptides. Pool 1: 23 peptides covering Ag85B residues 39 to 141; Pool 2: 23 peptides covering Ag85B residues 131 to 233; Pool 3: 23 peptides covering Ag85B residues 223 to 325.
- ESAT-6 was covered by 1 pool of 21 peptides. Pool 1: 21 peptides covering ESAT-6 residues 1 to 95.
- TB10.4 was covered by 1 pool of 21 peptides. Pool 1: 21 peptides covering TB10.4 residues 1 to 95.
- CFP-10 was covered by 1 pool of 23 peptides. Pool 1: 23 peptides covering CFP10 residues 1 to 99.
- Rv0287 was covered by 1 pool of 22 peptides. Pool 1: 22 peptides covering Rv0287 residues 1 to 96.

Individual peptides were resuspended in DMSO at 10 mM in average. Peptides were then pooled so that concentration of each peptide in each pool was 0.4 mM. For *ex vivo* stimulation, peptide pools were used at 1 µM final concentration. Resuspended peptide stocks and pools of peptides were stored at -20 °C.

**Triple Intracellular Cytokine Staining assay**

Splenocytes of each BLAB/c mouse were collected 7 days after one MVA injection. Red blood cells were then lysed (Sigma). Spleen cells (2.10^6^ /well) were seeded in flat-bottom 96-well plates and incubated at 37 °C in αMEM culture medium (Gibco BRL) supplemented with 10 % FCS (PAA), 80 U/mL penicillin + 80 µg/mL streptomycin (PAN), 2 mM L-glutamin (Gibco), 1X non-essential amino acids (Gibco), 10 mM Hepes (Gibco), 1 mM sodium pyruvate (Gibco) and 50 µM β-mercaptoethanol (Gibco), in presence of 1 μM of complete peptide pools for each antigen. A pool of peptides of β-gal was used as irrelevant stimulation. To monitor T cell responses specific of MVA vector, VGP (VGPSNSPTF) peptide belonging to MVA vector and restricted to H2-D^d^ was used. GolgiPlug (GP) was added to splenocyte after 1 h culture to block cytokine secretion. After a total of 6 h stimulation, cells were transferred into V-bottom 96-well plates and washed with 1 % FCS-PBS and incubated with 25 μL of anti-CD16/CF32 (clone 2.4G2) Fc Receptor block at a concentration of 2 μg/mL during 10 min at 4 °C. Then 25 μL of 1 % FCS-PBS containing Live/Dead Violet and monoclonal antibodies against CD4 (rat anti-mouse CD4 APC-H7, clone GK 1.5 at a concentration of 0.5 μg/mL), CD8a (rat anti-mouse CD8a V500, clone 53-6.7 at a concentration of 2 μg/mL) (both from BD Biosciences) were incubated 30 min at 4 °C. After washes, cells were fixed and permeabilized for 20 min in the dark at room temperature with Cytofix/Cytoperm, washed with Perm/Wash solution (BD Biosciences) and stored overnight at 4 °C. After washes, 50 μL of Perm/Wash solution containing monoclonal antibodies against CD3 (hamster anti-mouse CD3-PerCP, clone 145-2C11, at a concentration of 2 μg/mL), IFNγ (rat anti-mouse IFNγ-A488, clone XMG1.2, at a concentration of 2 μg/mL), IL2 (rat anti-mouse IL2-PE, clone JES6-5H4, at a concentration of 2 μg/mL), TNFα (rat anti-mouse TNFα-APC, clone MP6-XT22, at a concentration of 0.5 μg/mL) (all from BD Biosciences) were incubated 30 min at 4 °C. After washes, cells were resuspended in 200 μL with 1 % FCS-PBS and analysed by flow cytometry using a BD FACS Canto II cytometer. Two cut-off values were calculated. A technical cut-off value was determined as 25 x 100/average number of CD3e+, CD8α+ or CD4+ cells detected. An experimental cut-off value was also calculated as 3 times the standard deviation (SD) of values obtained with the medium condition (unstimulated condition). A response was then considered as positive if the percentage of cytokine-positive cell population was higher than the highest cut-off value between the technical and the experimental cut-off values.
