# Supplementary figures and images for: A multi-antigenic MVA vaccine increases efficacy of combination chemotherapy against Mycobacterium tuberculosis
Source: PLoS One. 2018 May 2;13(5):e0196815. doi: 10.1371/journal.pone.0196815 (PMC5931632; doi:10.1371/journal.pone.0196815)

## Slide 1
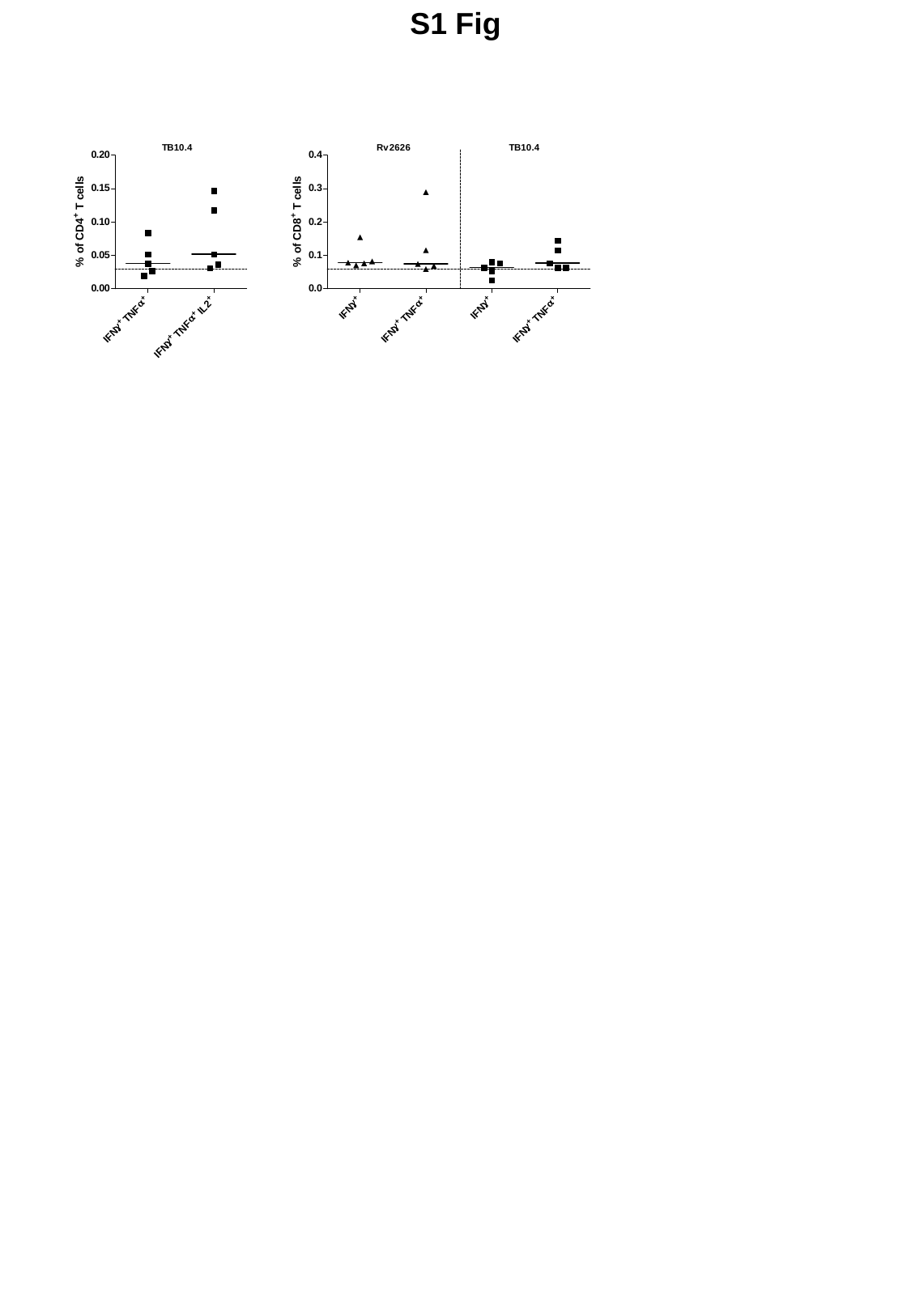

S1 Fig

Supplement: S1 Fig — Mice were immunized once with MVATG18598 or the empty vector MVATGN33.1, as a negative control. Cells were stimulated with Mtb peptide pools, the MVA vector-specific VGP peptide or an irrelevant E7 peptide. Results are presented as the percentage of single, double and triple positive CD8 or CD4 T cells for IFNγ, TNFα and IL2 among total CD4 and CD8 T cell populations, respectively. Each symbol represents response from individual mice and line represents median response. Cut-off values are indicated as dotted lines. Only cytokine-producing cell population above the cut-off values are represented. For each cell population, background signal obtained in unstimulated cells condition was subtracted. No cytokine-producing cell population was detected in mice vaccinated with MVATGN33.1 or following stimulation with the irrelevant E7 protein (not shown). Multi-cytokine-producing CD4 and CD8 T cells specific to the MVA vector were also detected in the MVATGN33.1-vaccinated control group as well as the MVATG18598-vaccinated group (data not shown). (PPTX) [file pone.0196815.s002.pptx]
