# Supplementary material for: A multi-antigenic MVA vaccine increases efficacy of combination chemotherapy against Mycobacterium tuberculosis
Source: PLoS One. 2018 May 2;13(5):e0196815. doi: 10.1371/journal.pone.0196815 (PMC5931632; doi:10.1371/journal.pone.0196815)
Supplement: S1 Table — Resuscit., Resuscitation. IFNγ ELISpot: group median value of spots detected using the following ranking: -, median < cut-off; +, 1x cut-off < median < 2x cut-off; ++, 2x cut-off < median < 3x cut-off; +++, 3x < median < 5x; ++++, median > 5x cut-off. CTL: -, no activity; +, CTL < 20%; ++, 20% < CTL < 30%). (PPTX) [file pone.0196815.s003.pptx]

## Slide 1
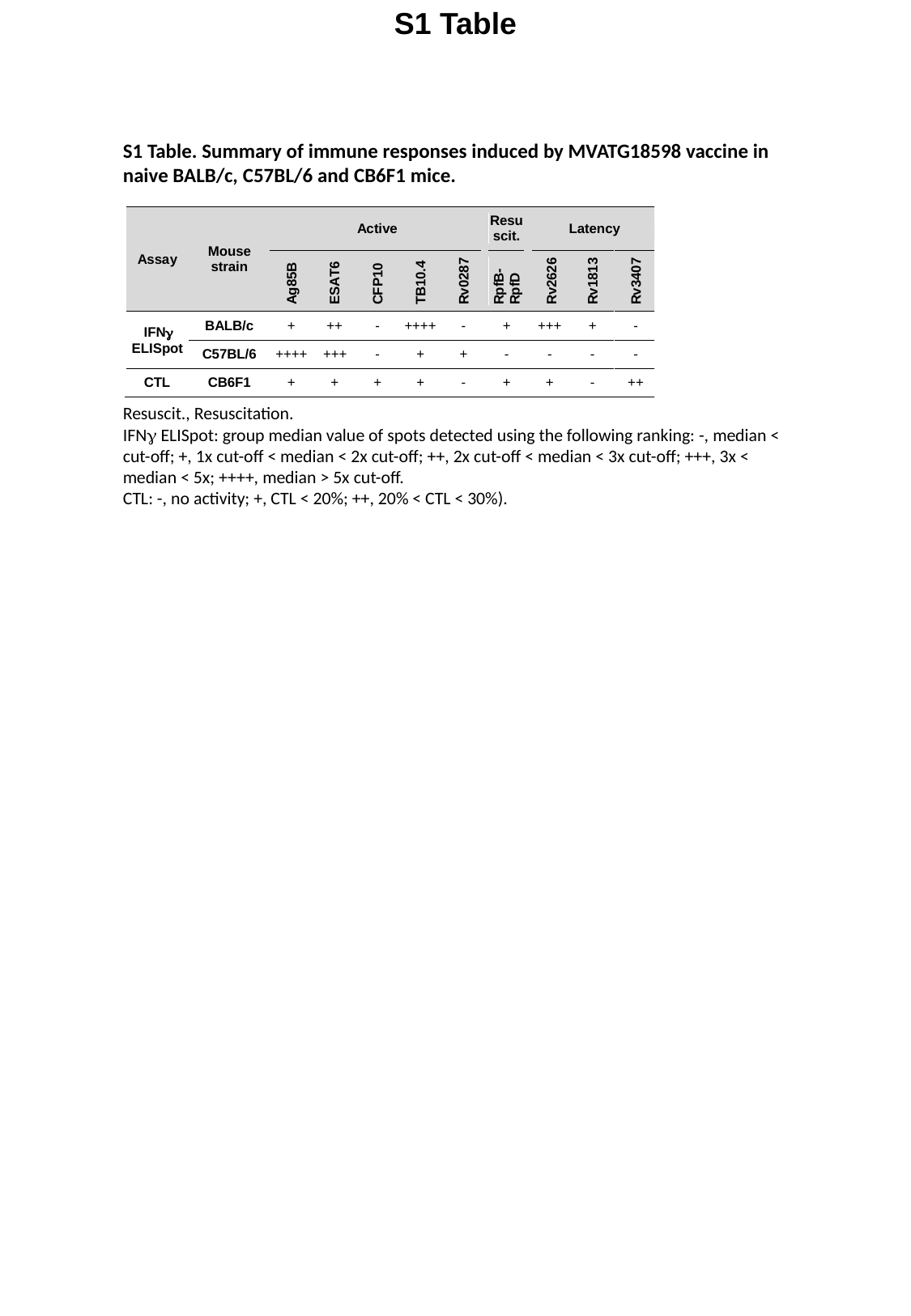

S1 Table
S1 Table. Summary of immune responses induced by MVATG18598 vaccine in naive BALB/c, C57BL/6 and CB6F1 mice.
Resuscit., Resuscitation.
IFNγ ELISpot: group median value of spots detected using the following ranking: -, median < cut-off; +, 1x cut-off < median < 2x cut-off; ++, 2x cut-off < median < 3x cut-off; +++, 3x < median < 5x; ++++, median > 5x cut-off.
CTL: -, no activity; +, CTL < 20%; ++, 20% < CTL < 30%).
